# Supplementary material for: ciBAR1 loss in mice causes laterality defects, pancreatic degeneration, and altered glucose tolerance
Source: Life Sci Alliance. 2024 Dec 2;8(2):e202402916. doi: 10.26508/lsa.202402916 (PMC11612972; doi:10.26508/lsa.202402916)
Supplement: Supplementary file 1 [file LSA-2024-02916_TableS1.docx]

| **Name** | **Species** | **Manufacturer** | **Catalog number** | **RRID** | **Dilution** |
| --- | --- | --- | --- | --- | --- |
| Acetylated α-tubulin | Mouse | Sigma-Aldrich | T7451 | AB_609894 | 1:200 |
| γ-Tubulin (GTU-88) | Mouse | Sigma-Aldrich | T6557 (079K4861) | AB_477584 | 1:200 |
| CD45 (D3F8Q) | Rabbit | Cell Signaling | 70257 | AB_2799780 | 1:400 |
| Cby1 | Mouse | In-house (Voronina et al., 2009) | N/A | N/A |  |
| ciBAR1 | Rabbit | Proteintech | 24803-1-AP | AB_2879735 | 1:200 |
| ciBAR2 | Rabbit | Proteintech | 26413-1-AP | AB_2880506 | 1:200 |
| Insulin (human) | Guinea pig | Linco (Millipore) | 4011-01F (HI01F-K) | AB_433703 | 1:200 |
| Glucagon | Mouse | Proteintech | 67286-1-Ig | AB_2882552 | 1:200 |
| Somatostatin (64-81aa) | Rabbit | Proteintech | 17512-1-AP | AB_2195910 | 1:200 |
| Centrin 1 | Rabbit | Proteintech | 12794-1-AP | AB_2077371 | 1:100 |
| ARL13B | Mouse | Proteintech | 17711-1-AP | AB_2060867 | 1:200 |
| Dolichos Biflorus Agglutinin (DBA), Fluorescein | N/A | Vector Laboratories | FL-1031 | AB_2336394 | 1:200 |
| Peanut Agglutinin (PNA), Rhodamine | N/A | Vector Laboratories | RL-1072-5 | AB_2336642 | 1:200 |
| Anti-Mouse IgG2b AF 555 | Goat | ThermoFisher | A21147 | AB_2535783 | 1:500 |
| Anti-Mouse IgG1 AF488 | Goat | Invitrogen | A21121 | AB_2535764 | 1:500 |
| Anti-Rabbit CoraLite 488 | Goat | Proteintech | SA00013-2 | AB_2797132 | 1:500 |
| Anti-Mouse AF555 PLUS | Goat | Invitrogen | A32727 | AB_2633276 | 1:500 |
| Anti-Rabbit AF488 PLUS | Goat | Invitrogen | A32731 | AB_2633280 | 1:500 |
| Anti-Guinea Pig ML DL488 | Donkey | Jackson ImmunoResearch Labs | 706-485-148 | AB_2617153 | 1:500 |
| Anti-Rabbit AF647 | Goat | Invitrogen | A21244 | AB_2535812 | 1:500 |
| Anti-Mouse IgG DyLight488 | Goat | Jackson ImmunoResearch Labs | 115-485-146 | N/A (discontinued) | 1:500 |
